# Prevalence, Pattern and Associated Risk Factors of Dry Eye Disease From a Prospective Database of a Tertiary Eye Care Centre in Central India

**DOI:** 10.7759/cureus.78889

**Published:** 2025-02-12

**Authors:** Sunita Sabarwal, Priyanka, Bruttendu Moharana, Rituka Gupta, Bhavana Sharma

**Affiliations:** 1 Ophthalmology, All India Institute of Medical Sciences, Bhopal, Bhopal, IND; 2 Ophthalmology, All India Institute of Medical Sciences, Bhubaneswar, Bhubaneswar, IND

**Keywords:** central india, dry eye disease, pattern, prevalence, risk factors

## Abstract

Purpose

This study aimed to assess the prevalence, pattern and associated risk factors with dry eye disease (DED) at a tertiary eye care centre in Central India.

Methods

This prospective cross-sectional study, conducted over 48 months, enrolled patients with systematic random sampling. Evaluation of DED was done with subjective and objective methods comprising of the Ocular Surface Disease Index (OSDI) questionnaire, slit lamp examination, Schirmer’s tests and fluorescein tear-film breakup time (FTBUT) test. Patients were categorised into mild, moderate, severe and very severe DED. Association with various etiological factors like age, sex, occupation, diabetes mellitus, autoimmune disorders and any other systemic illness was assessed. The chi-square test/Fischer's exact test was used to analyse categorical data. Bivariate logistic regression analysis was used to calculate the odds ratio (OR). Multivariate analysis was performed to identify independent risk factors.

Results

A total of 2,560 patients were evaluated, of which 640 patients (25%) had DED. Prevalence was higher in patients aged >50 years, household workers, students, government employees and farmers. Uncorrected refractive status, cigarette smoking, ocular allergy and contact lens usage were associated with increasing odds of developing DED.

Conclusion

The prevalence of DED was estimated to be 25% in central India. Certain occupations like household workers, students, government employees, farmers, uncorrected refractive status, cigarette smoking, ocular allergy and contact lens use were significant risk factors for dry eyes.

## Introduction

Dry eye disease (DED) is a multifactorial sight-threatening entity with ocular surface inflammation consequent to tear film instability, hyperosmolarity, trophic damage and neurosensory abnormalities [1]. It is the most frequent disorder in ophthalmology practice. The prevalence of dry eyes ranges from 10.8% to 57.1%, showing a wide variation in demography, ethnicity, geographical location, climatic factors and occupation. Such disparity can also occur due to non-standardized criteria for patient selection, diagnosis and stratification of dry eyes [2]. Epidemiologic studies are helpful in the determination of causes and prevalence and help devise appropriate measures for prevention and treatment.

This study attempts to concentrate on the most recent information on the epidemiology of DED. Although many studies on the epidemiological pattern of DED have been reported from different parts of the world, very few studies are available from India [2-6]. To the best of our knowledge, this is the first reported prospective series of disease prevalence, pattern and associated risk factors of DED from Central India. The primary objective of this study was to evaluate the prevalence of dry eye disease at a tertiary eye care centre and to study the pattern, predisposing causes and risk factors associated with dry eye disease at a major tertiary eye centre in central India, which caters to around 30 lakh population of the district and is the tertiary referral centre for the entire state. DED accounted for around 1% of our hospital-based daily ophthalmological outpatient visits, around two to three per day.

## Materials and methods

A hospital-based cross-sectional study was conducted at the outpatient services of the department in All India Institute of Medical Sciences, Bhopal, a tertiary eye care centre in Central India. The study duration was 48 months and was approved by Research and Review Board (RRB) and the Institutional Human Ethics Committee (IHEC) (approval no. IHEC-LOP/2019/MD0065). The study adhered to the tenets of the Declaration of Helsinki. Patients attending the outpatient services of the department were selected by systematic random sampling. Every 15th patient on the three OPD days per week was enrolled in the study. The next patient was taken if the selected subject was unwilling to participate in the study. Subjects unwilling to participate in the study, aged <10 years, patients with acute infection, those with undiagnosed systemic disorders requiring treatment and patients with incomplete medical records were excluded from the study.

Written informed consent was obtained from all the participants/parents or guardians of minor subjects. Detailed ocular history was recorded with reference to the onset, severity, laterality, duration, progression of symptoms and nature of the disease. History of any associated pain, irritation, redness and burning/stinging, tearing, contact lens intolerance, increased frequency of blinking, itchy eyes, foreign body sensation, blurred vision, photophobia and thick sticky mucous discharge was taken. A detailed evaluation of systemic signs and symptoms was done to look for other associated autoimmune or connective tissue diseases. 

A slit lamp examination of the lid was done for the presence of any anatomical abnormalities, blepharitis, vascularisation or foreign body. Meibomian orifices were examined for pouting, presence of foam, secretion and plugging. Tarsal conjunctiva was examined for papillae, congestion, symblepharon, conjunctival xerosis and scarring.

The cornea was examined to evaluate the epithelial defect, conjuctivalisation, limbal ischemia, vascularisation and dystrophy or degeneration. Each recruited subject was investigated using the Ocular Surface Disease Index (OSDI) [7]. A single observer completed the questionnaire, and the questionnaire was explained in their local language for those who were not conversant in English. The objective test was done in patients with OSDI scores >10 by a single examiner in the same room to maintain similar temperature and humidity conditions for all patients. Schirmer’s test I and II and fluorescein tear-film breakup time (FTBUT) were done, and patients were graded into mild, moderate, severe and very severe DED under the Tear Film and Ocular Surface Society Report of the TFOS International Dry Eye Workshop (TFOS DEWS II) [8].

Statistical analysis

Statistical analyses were performed using IBM SPSS Statistics for Windows, Version 22.0 (released 2013, IBM Corp., Armonk, NY). The chi-square test, Fischer exact test and linear regression analyses were used to establish the association between categorical data. Bivariate logistic regression analysis was used to calculate the odds ratio (OR). Multivariate analysis was performed to identify independent risk factors. To identify factors related to the predisposition of disease, P values of 0.05 or less within 95% CI were considered to be statistically significant.

## Results

A total of 2,560 subjects were enrolled in this study; 1,396 (54.53%) were male and 1,164 (45.47%) were female (M: F = 1.19:1). The mean age of the study participants was computed to be 48.1 ± 19.5 years. The study population was divided into four groups as per their age, i.e., 10-30 years, 31-50 years, 51-70 years and above 70 years. The demographic details of the patients are summarised in Table 1.

**Table 1:** Demographic details of patients with dry eye disease (DED)

Characteristic	Total no of patiens (%)	No. of patients with DED	% Prevalence
Age(years)			
10-30	627 (24.5%)	167	26.7%
31-50	658 (25.7%)	197	29.9%
51-70	648 (25.3%)	232	35.8%
>70	627 (24.5%)	44	7%
Gender			
Male	1396 (54.53%)	308	22.1%
Female	1164 (45.47%)	332	28.5%
Place of residence			
Urban	1485 (57.97%)	340	22.9%
Rural	1075 (42.03%)	300	27.9%

Out of the 2,560 study subjects, 640 (25%) were diagnosed as having DED. Patients diagnosed with DED were categorised further into severity grades as mild (240, 37.5%), moderate (252, 39.37%), severe (120, 18.75%) and very severe DED (28, 4.37%). The maximum prevalence of DED was found among the 51-70 years age group (232/648; 35.8%), followed by the 31-50 years age group (197/658; 29.9%), 10-30 years age group (167/627; 26.7%) and above 70 years age group (44/627; 7%). The association was highly significant, with a P-value of 0.0001. However, the association between age and disease severity was not found to be statistically significant (P = 0.1). The prevalence of DED was observed to be 27.9% (300/1075) among the rural population, while it was 22.9% (340/1485) among the urban population, which was statistically insignificant (P = 0.2).

The most common symptom of DED was observed to be watering (235, 36.8%), followed by redness (160, 25%), gritty sensation (128, 20%) and burning sensation (120, 18.7%). Itching and blurring of vision were observed in 84 (13.1%) and 43 (6.8%) of the subjects, respectively.

The prevalence of DED was higher among the female subjects (332/1164, 28.5%) than the male subjects (308/1396, 22.1%), but the association was not found to be significant statistically (p = 0.07). Both the groups were further analysed for association with the severity of the disease. Among males, a maximum number of cases were found in the mild category (116), while among females, the maximum cases were under the moderate category (148). In both genders, the minimum number of cases were seen in the very severe disease category, i.e., 20 and 8 in males and females, respectively. On statistical analysis, the correlation between gender and disease severity was not significant (P = 0.42).

Prevalence was seen to be maximum among the household workers (232/640, 36.2%), followed by mobile and computer user students (116/640, 18.1%), government employees (104/640, 16.2%) and farmers (80/640, 12.5%) (Table 2).

**Table 2:** Prevalence and severity of dry eye disease as correlated to the occupation of the patients.

Occupation	Mild	Moderate	Severe	Very severe	Total	% (N = 160)
Farmer	24	36	12	8	80	12.5
Teacher	16	0	4	0	20	3.125
Tailor	0	24	16	4	44	6.9
Software worker	0	16	4	0	20	3.1
Labourers	4	0	12	0	16	2.5
House Hold worker	100	96	36	0	232	36.25
Factory Worker	4	0	4	0	8	1.25
Student (mobile users)	40	56	16	4	116	18.1
Govt Employee (computer work)	52	24	16	12	104	16.2
TOTAL	240	252	120	28	640	100

A severe form of the disease was prevalent more amongst household workers and students (mobile users), government employees, tailors and farmers. The probability of very severe DED was more common in government employees, farmers, tailors and students. The association between occupation and disease severity was statistically significant (P = 0.02).

Concerning refractive errors, the prevalence of DED was computed to be a maximum of 31.8% among uncorrected refractive errors, 22.3% among corrected refractive errors and 20.6% among emmetropes. The association between refractive status and DED occurrence was statistically significant (P = 0.02).

 In patients who were diagnosed as having DED, 14.4% (92/640) of the subjects were shown to have various systemic comorbidities. Forty (6.2%) patients had type 2 DM, 16 (2.5%) had hypertension, 12 (1.9%) had rheumatoid arthritis, 16 had (2.5%) thyroid disease and five (1.2%) had Sjogren syndrome.

The study subjects were assessed for any risk factors associated with DED. Major risk factors observed were near work (students, mobile users), computer work, post-menopause and smoking, which constituted approximately 70% of DED patients. A bivariate analysis of the risk factors associated with the development of severe DED and a multivariate analysis of risk factors associated with DED was undertaken (Table 3).

**Table 3:** Bivariate and multivariate analysis of factors associated with the severity of dry eye disease.

Risk Factor	Severe DED	Non-severe DED	P	Unadjusted OR (95% CI)	Adjusted OR (95% CI)
Smoking	48	36	<0.001	1.5 (0.6-3.4)	1.79 (0.93-3.4)
Alcohol consumption	0	28	0.18	0.4 (0.19-1.0)	2.5 (2.1-2.9)
Post-ocular surgery	4	28	0.88	1.31 (0.5-3.1)	1.89 (1.10-3.51)
Post-menopause	8	88	0.20	1.17 (0.9-1.5)	2.3(1.9-2.8)
Pterygium	0	16	0.13	0.80 (0.7-1.1)	1.6(.9-1.8)
Ocular allergy	32	16	<0.001	1.5 (1.19-1.88)	2.14 (1.6-2.7)
Topical medication	4	40	0.50	2.4 (1.90-3.66)	3.5 (2.40-5.72)
Post-viral keratitis	0	12	0.29	1.89 (1.5-2.90)	2.14 (1.6-2.7)
Systemic illness	16	76	0.19	1.21(1.1-1.22)	2.32(1.11-2.83)

Significant odds of having severe DED were associated with smoking, contact lenses and ocular allergy. Cigarette smoking (P < 0.001, OR 1.5; 95% CI 0.6-3.4), ocular allergy (P < 0.001, OR 1.5; 95% CI 1.19-1.88) and contact lens usage (P < 0.001, OR 6.4; 95% CI 3.3-12.6) were identified as significant risk factors for severe DED. On logistic multivariate analysis, we did not observe any significant association between more than one coexistent risk factor and the occurrence of DED.

## Discussion

Dry eye is a multifactorial disease of the ocular surface characterised by a loss of tear film homeostasis. The accompanying ocular symptoms are caused by tear film instability, hyperosmolarity, ocular surface inflammation and neurosensory abnormalities. The prevalence of DED varies with the operational definition and the characteristics of the population studied. Early detection and timely treatment are of immense importance in managing DED. In different parts of the world, varying prevalence patterns are most likely due to variations in geographic, genetic, sociodemographic and treatment-related factors. This study is intended to evaluate the prevalence and associated risk factors in a hospital-based setting and to suggest preventive measures and timely management of DED, which will further reduce the burden of blindness and morbidity associated with it. Out of the 2560 study subjects, 640 (25%) were diagnosed with dry eye. The lower prevalence of 25% in the present study is contrary to 32% and 34.2%, as reported by Titiyal et al. and Shilpy et al. [4,9] These studies relied on only symptoms to estimate the prevalence of DED, which may have resulted in an overestimation of the prevalence of DED. The prevalence varies with subjects’ geographical location, environmental conditions and lifestyle; hence, places with extreme temperatures and dry weather conditions report a higher prevalence of DED. Factors relating to lifestyle, like sitting in air-conditioned rooms, using digital devices and smoking, increase the likelihood of DED. Past studies suggest that dry eye prevalence ranges from 10.8% to 57.1 % [2]. Such disparity can be attributed to a lack of standardisation of dry eye diagnostic criteria and cut-off values for diagnostic tests.

Association with age

The prevalence of DED in different age groups is important in determining the disease burden in categorised subjects. The prevalence of DED is known to increase with age. The maximum prevalence of DED (35.8%) was found to be in the 51-70 years age group. The difference was statistically significant. These results agreed with studies by Attri et al., Donthineni et al. and Mathers et al., which reported that the prevalence of dry eye was higher in higher age groups [10-12]. This is likely a result of a pathological decrease in tear production and tear stability associated with advancing age and an age-related decrease in meibomian gland secretion due to atrophy of acinar cells [13]. By contrast, the severity of DED had no significant association with different age groups.

The lowest prevalence (7%) was in the extreme age group (patients >70 years). In the >70-year age group, the prevalence of DED was paradoxically less as compared to increased prevalence in patients in the younger age group. We also found significantly less frequency of associated risk factors comprising VDT use, electronic gadgets, and mobile phones in this age group compared to the younger age group, which could be the possible explanation for decreased prevalence in the >70-year-old age group.

Association with gender

Although the prevalence of DED was higher in females, the difference was not statistically significant. These results are similar to the study done by Sherry et al. [14]. Contrary to these findings, a significant association between gender and the prevalence of DED have been reported by Attri et al., Moss et al. and Bakkar et al. [10,15,16]. A study by Moss et al. reported a lower prevalence in men than women and was explained by the effect of hormonal loss during menopause on the ocular dryness of women [15]. Furthermore, the severity of DED was not significantly associated with the gender of the studied population, which indicated a lack of temporal association between disease severity and gender. This could be due to a higher proportion of non-elderly subjects in our study. Elderly females and rural populations living in far areas are less likely to visit hospitals due to sociocultural factors, which could be potential causes of possible decreased prevalence.

Association with locality

The association between the prevalence of DED and residential location (rural vs urban) was found to have a non-significant correlation. These results contrast with the findings of studies done by Donthineni et al., Um et al. and Moon et al. [11,17,18]. However, our study is similar to Alshamrani et al. [19]. This can be attributed to the use of electronic gadgets and visual display terminals (VDTs) in equal frequency among both rural and urban populations in India.

Association with occupation

The prevalence of disease was observed to bear a significant association with the occupation of studied subjects. The maximum prevalence of DED was in household workers, followed by students and government employees. Maximum cases of very severe type DED were found in government employees engaged with computer work. Most of the household workers belonged to rural and low socioeconomic backgrounds; hence, elderly females had a history of exposure to smoke and dust while cooking food on “chulha”. Due to their low socioeconomic background, they were deprived of good dietary intake containing essential nutrients. Frequent use of electronic gadgets, visual display terminal (VDT) use and air-conditioned atmosphere creating dry conditions could be the reason for urban and patients with higher socioeconomic backgrounds [20]. Findings similar to our results have been reported in other studies [4,11,21,22].

Association with the refractive status

The prevalence of DED was found to be significantly associated with refractive status and was highest in uncorrected refractive error (31.8%), followed by corrected refractive error (22.3%) and emmetropes (20.6%). Sahai et al. have reported a similar high incidence of DED in patients with refractive error [2.] It is postulated that persons with refractive errors have an increased tendency to rub their eyes, which could cause the lodgment of particulate foreign substances, contaminants, sebum and sweat into the eye, predisposing to tear film instability [15]. This result contrasts with Gupta et al., who reported that refractive error status does not affect the occurrence of DED [3]. Patients more than 50 years of age group had a coexistent refractive error, accounting for the increased prevalence of DED in the referenced age group. Meanwhile, in younger populations, the history of use of electronic gadgets was significantly high, so refractive status could be a confounding factor to these already proven risk factors.

Association with systemic illnesses

Dry eye has been associated with systemic conditions, including arthritis, diabetes and thyroid disease. In the present study, 14.4% of the total 640 DED patients were found to have associated illnesses such as type 2 DM, thyroid disease, rheumatoid arthritis and Sjogren’s syndrome. Elevated levels of circulating proinflammatory cytokines and inflammatory cell infiltration of lacrimal glands and associated MGD are responsible for ocular involvement in systemic illness. Many studies reported an association between DED and similar risk factors [15, 23-25].

Risk factors

DED has a multifactorial etiopathogenesis. Cigarette smoking, ocular allergy and contact lens usage were identified as significant risk factors for DED. There was no significant association between severe DED and a history of previous ocular surgery, post-menopause females, alcohol consumption, topical medications, post-viral keratitis and pterygium and systemic illness.

Contact lens usage may cause dry eye or aggravate preexisting DED. Contact lenses cause meibomian gland dysfunction, corneal hyposensitivity or a combination, which results in decreased tear production, increased evaporation and subsequent tear film hyperosmolarity [26]. Other studies reported similar results [4,15,27].

Smoking may affect the tear film stability and ocular surface sensitivity and act as a direct irritant to the eyes [15]. A significant association has been reported between smoking and DED in many other studies, too [4,15,28].

The Tear Film and Ocular Surface Society Dry Eye Workshop II recently included allergic conjunctivitis among the "probable" risk factors for DED [8,29]. Recent advances in the DED pathogenic mechanisms provide a strong rationale for considering ocular allergy as a risk factor for DED [30]. Ocular allergy is associated with releasing inflammatory mediators in tears, which can damage surface epithelium and cause tear film instability.

To our knowledge, this is the first large sample size study from a tertiary eye care centre in central India to ascertain epidemiological patterns and risk factors for DED. It incorporates subjective tools with objective clinical tests to determine dry eye prevalence and risk factors. Most epidemiological studies conducted earlier included the elderly population (>40 years of age), which falls short of representing the actual disease prevalence in the referenced population. With the increasing use of electronic gadgets by the younger age group, there is a need for more epidemiological studies that widen their inclusion criteria to ascertain precise estimates of the prevalence of DED. This study was designed and conducted to bridge this significant gap in knowledge effectively. As this study is a hospital-based cross-sectional study, there are some limitations in extrapolating the results to the general population of this region. Females, the elderly and the rural population living in far areas are less likely to visit hospitals due to the sociocultural environment. This could be a potential cause of decreased prevalence. The study's cross-sectional design precludes us from knowing the antecedent-consequent relationship between risk factors and the endpoint.

## Conclusions

The prevalence of DED was estimated to be 25%, with the most commonly affected age group of 51-70. Age and occupation of study subjects were directly significantly correlated with the occurrence of DED. The maximum prevalence of DED was found in household workers, students and government employees. Uncorrected refractive status was significantly associated with the prevalence of DED. We did not observe any significant association between age, gender and place of residence with the occurrence of DED. Concerning risk factors, cigarette smoking, ocular allergy and contact lens usage were associated with increasing odds of developing DED. Extensive data evaluation supported by adequate sample size and statistical correlation collates this study to represent the prevalence of DED in central India.

This study also provides new insights into the magnitude, risk factors and causes of DED across all age groups and is the pioneer documentation to analyse the epidemiology and aetiology in central India.

## References

[1] Chao W, Belmonte C, Benitez Del Castillo JM (2016). Report of the inaugural meeting of the TFOS i(2) = initiating innovation series: targeting the unmet need for dry eye treatment. Ocul Surf.

[2] Sahai A, Malik P (2005). Dry eye: prevalence and attributable risk factors in a hospital-based population. Indian J Ophthalmol.

[3] Gupta N, Prasad I, Jain R, D'Souza P (2010). Estimating the prevalence of dry eye among Indian patients attending a tertiary ophthalmology clinic. Ann Trop Med Parasitol.

[4] Titiyal JS, Falera RC, Kaur M, Sharma V, Sharma N (2018). Prevalence and risk factors of dry eye disease in North India: Ocular surface disease index-based cross-sectional hospital study. Indian J Ophthalmol.

[5] Shah S, Jani H (2015). Prevalence and associated factors of dry eye: our experience in patients above 40 years of age at a tertiary care center. Oman J Ophthalmol.

[6] Basak SK, Pal PP, Basak S, Bandyopadhyay A, Choudhury S, Sar S (2012). Prevalence of dry eye diseases in hospital-based population in West Bengal, Eastern India. J Indian Med Assoc.

[7] Schiffman RM, Christianson MD, Jacobsen G, Hirsch JD, Reis BL (2000). Reliability and validity of the Ocular Surface Disease Index. Arch Ophthalmol.

[8] (2007). The definition and classification of dry eye disease: report of the Definition and Classification Subcommittee of the International Dry Eye WorkShop (2007). Ocul Surf.

[9] Shilpy N, Patel DB (2019). Prevalence of dry eye disease in Western India. Int J Contemp Med Res.

[10] Attri S, Dwivedi J, Mithal S, Gupta A, Singh LK (2019). Dry eye-study of prevalence, associated risk factors and frequency of symptoms in Meerut District. J Evol Med Dent Sci.

[11] Donthineni PR, Kammari P, Shanbhag SS, Singh V, Das AV, Basu S (2019). Incidence, demographics, types and risk factors of dry eye disease in India: electronic medical records driven big data analytics report I. Ocul Surf.

[12] Mathers WD, Lane JA, Zimmerman MB (1996). Tear film changes associated with normal aging. Cornea.

[13] Adlakha N, Tirkey ER, Lakhtakia S (2017). To assess the prevalence of dry eye disease in postmenopausal females in a tertiary care centre in Central India. J Med Sci Clin Res.

[14] Sherry A, Aridi M, Ghach W (2020). Prevalence and risk factors of symptomatic dry eye disease in Lebanon. Cont Lens Anterior Eye.

[15] Moss SE, Klein R, Klein BE (2000). Prevalence of and risk factors for dry eye syndrome. Arch Ophthalmol.

[16] Bakkar MM, Shihadeh WA, Haddad MF, Khader YS (2016). Epidemiology of symptoms of dry eye disease (DED) in Jordan: a cross-sectional non-clinical population-based study. Cont Lens Anterior Eye.

[17] Um SB, Kim NH, Lee HK, Song JS, Kim HC (2014). Spatial epidemiology of dry eye disease: findings from South Korea. Int J Health Geogr.

[18] Moon JH, Kim KW, Moon NJ (2016). Smartphone use is a risk factor for pediatric dry eye disease according to region and age: a case control study. BMC Ophthalmol.

[19] Alshamrani AA, Almousa AS, Almulhim AA (2017). Prevalence and risk factors of dry eye symptoms in a Saudi Arabian population. Middle East Afr J Ophthalmol.

[20] Bazeer S, Jansonius N, Snieder H, Hammond C, Vehof J (2019). The relationship between occupation and dry eye. Ocul Surf.

[21] Lee JH, Lee W, Yoon JH, Seok H, Roh J, Won JU (2015). Relationship between symptoms of dry eye syndrome and occupational characteristics: the Korean National Health and Nutrition Examination Survey 2010-2012. BMC Ophthalmol.

[22] Rahman ZA, Sanip S (2011). P2-493 computer vision syndrome: the association with ergonomic factors. J Epidemiol Community Health.

[23] Khurana G, Khurana D, Jain R (2016). Dry eye in patients with diabetic retinopathy: a clinical study. Delhi J Ophthalmol.

[24] Kumar A, Vyas M (2018). Dry eye and corneal sensitivity in patients with type 2 diabetes mellitus. Int J Med Sci Clin Res.

[25] Lee CY, Chen HC, Sun CC (2019). Gout as a risk factor for dry eye disease: a population-based cohort study. J Clin Med.

[26] Chen SP, Massaro-Giordano G, Pistilli M, Schreiber CA, Bunya VY (2013). Tear osmolarity and dry eye symptoms in women using oral contraception and contact lenses. Cornea.

[27] Paulsen AJ, Cruickshanks KJ, Fischer ME, Huang GH, Klein BE, Klein R, Dalton DS (2014). Dry eye in the beaver dam offspring study: prevalence, risk factors, and health-related quality of life. Am J Ophthalmol.

[28] Xu L, Zhang W, Zhu XY, Suo T, Fan XQ, Fu Y (2016). Smoking and the risk of dry eye: a meta-analysis. Int J Ophthalmol.

[29] Stapleton F, Alves M, Bunya VY (2017). TFOS DEWS II Epidemiology Report. Ocul Surf.

[30] Villani E, Rabbiolo G, Nucci P (2018). Ocular allergy as a risk factor for dry eye in adults and children. Curr Opin Allergy Clin Immunol.

